# “Something we can all share”: Exploring the social significance of food insecurity for young people in KwaZulu-Natal, South Africa

**DOI:** 10.1371/journal.pgph.0003137

**Published:** 2024-05-28

**Authors:** Laura J. Brown, Jenevieve Mannell, Laura Washington, Sivuyile Khaula, Andrew Gibbs

**Affiliations:** 1 Institute for Global Health, University College London, London, United Kingdom; 2 Project Empower, KwaZulu-Natal, South Africa; 3 Department of Psychology, University of Exeter, Exeter, United Kingdom; 4 Gender and Health Research Unit, South African Medical Research Council, Pretoria, South Africa; 5 Centre for Rural Health, University of KwaZulu-Natal, Durban, South Africa; PLOS: Public Library of Science, UNITED STATES

## Abstract

Food insecurity remains a pressing global issue and South Africa continues to face socioeconomic inequalities that make securing food a challenge for many young people. To address this challenge, we need better understanding of the social context of food and its importance in driving perceptions and behaviours about food and its scarcity. In this study, we examine the meaning of food for young people living in urban informal settlements and rural villages in KwaZulu-Natal, and investigate how they exert agency in the face of food insecurity. We use qualitative data from 17 photo/video elicitation interviews conducted from December 2020-January 2021 with young people experiencing food insecurity. The sample consisted of 9 women and 8 men who were part of the *Siyaphambili Youth ("Youth Moving Forward")* project. Data were analysed using thematic analysis. Themes included the challenges young people face in securing food and money for food. However, in exploring young people’s agency, food also plays a critical role in shaping their identities and social networks. Relevant themes included the use of food as a means of bonding with others; solidifying relationships; and as a signifier of social status and gender roles. Despite the challenges of food insecurity, young people demonstrated resilience and agency, utilising social and gendered coping strategies to secure food and to maintain their social networks. Our study contributes to the understanding of food insecurity amongst young people in South Africa and highlights the need for a comprehensive and culturally sensitive approach to addressing this issue. We argue that interventions aimed at addressing food insecurity should prioritise the empowerment of local communities and consider the sociocultural and gendered context of food in their design and implementation.

## Background

Food is essential for human survival, providing necessary nutrition and sustenance for physical and mental wellbeing [1]. The United Nations Sustainable Development Goals (SDGs) recognise food security as a crucial aspect of sustainable development (Goal 2), highlighting the importance of eradicating hunger, achieving food security, and promoting sustainable agriculture by 2030 [2]. The availability of adequate and nutritious food is a fundamental human right, yet food insecurity remains a significant challenge worldwide; over 690 million people experienced chronic hunger and nearly one in ten people in the world experienced severe food insecurity in 2019 [3]. The problem is particularly acute in regions that are already facing significant environmental challenges, such as climate change and water insecurity [4–7]. This issue is particularly acute in sub-Saharan Africa, where food insecurity affects an estimated third of the population [1]. In this paper, we explore the experiences of young people living in South Africa, in relation to food insecurity and the social context of food.

In an era of increasing commitment to alleviating food insecurity [8], including for marginalised groups such as women, young people and the unemployed, there is surprisingly still very limited attention to the structural causes of food insecurity [9]. This obscures the broader social and political context in which food insecurity is experienced. It is important to recognise that food insecurity is not just about a lack of food; it is also about power, inequality, and social exclusion [10]. Food serves as a language through which to explore stories about belonging, sexuality, fulfilment, and freedom [9]. By exploring the social context of food, we can gain a deeper understanding of how structural factors such as gender, race, and class intersect with food access and influence individuals’ experiences of food insecurity.

Gender is a particularly salient factor in understanding the social context of food and food insecurity. For example, traditionally, in many cultures, masculinity is equated to the provision of food while femininity is equated to the preparation of food [10–13]. By examining the gendered dimensions of food insecurity, we can better understand the ways in which social structures and cultural norms shape access to food and develop interventions that address these underlying drivers of food insecurity. A feminist approach calls for more research exploring relationships with food at global, macro and micro levels and that centres lived experiences [9]. Such an approach also calls for a move away from simply identifying food inequalities and its victims, and instead towards a focus on agency [9]. We need to listen to the voices of local people who are forging their own survival strategies in the face of food insecurity. Simultaneously, the symbolic value of food needs appreciating to fully understand food security issues: encounters with food far exceed the simple processes of consumption and nutrition [9].

Drawing on a feminist approach, we examine food insecurity through the conceptual lens of young people’s agency. The concept of agency refers to an individual or collective’s ability to make choices and take action, despite being in a situation of constraint or limitation [14,15]. It is the capacity of individuals or groups to act purposefully and make decisions that influence their own lives and the lives of those around them. In the context of food insecurity, agency can be seen as a vital factor in how people respond to and cope with food scarcity [16]. By recognising the agency of young people in the face of food insecurity, we can better understand how they navigate their social and economic environments, and develop interventions that build on their existing strengths and resources. As a result, this study focuses on the meaning of food for young people, how they experience food insecurity, and the ways in which they exert agency in the face of food insecurity.

### Study setting

South Africa experiences considerable socio-economic challenges, including high levels of poverty, unemployment, and inequality [17,18], with significant disparities in food access and food security across the country. Almost a quarter (23.6%) of people living in South Africa in 2020 were affected by moderate or severe food insecurity, while almost 14.9% experienced severe food insecurity [19]. South Africa’s recent trajectory of low economic growth and increasing unemployment, especially after COVID-19, is a significant hurdle in addressing food insecurity [19]. The Government of South Africa has been providing a Social Relief of Distress (SRD) Grant of R350 per month since April 2022 for people who are unemployed [20], but the Treasury have been making fewer funds available, meaning that more people are being excluded; in 2022, 4.65 million food-insecure people had still not received this government funding [21]. This challenging economic situation means that levels of hunger are likely to remain high or even increased in the aftermath of COVID [22].

KwaZulu-Natal (KZN), one of South Africa’s most populous provinces, faces particular challenges around food insecurity, with data from 2016 suggesting that 23.3% of households didn’t have money to buy enough food in the past 12 months [23]. This in the context of a poverty headcount of 7.7% and an intensity of poverty at 42.5%, although these measures of poverty vary substantially within the province, with some districts reaching as high as 15.7% and 44.1%, respectively [23]. 2021 data suggests a similar pattern, with 18.6% of KZN households experiencing food insecurity, and 14.3% being at risk [24]. Many rely on social grants as their primary source of income [25]. Young people in KZN’s urban informal settlements and rural villages are especially vulnerable to food insecurity [26,27]. Poverty begins in childhood, with more than three quarters of children in KZN living below the poverty line [25]. Growing up in these challenging conditions, young people are then faced with limited access to adequate nutrition and few economic opportunities to improve their situations [28].

### Overarching research question

What does food mean to young people in urban informal settlements and rural villages in KwaZulu-Natal and how do young people exert agency in the face of food insecurity?

## Methods

### Ethics statement

Ethical approval for the development of the *Siyaphambili Youth* intervention was granted by the South African Medical Research Council (SAMRC) (EC041-10/2020) and University College London (UCL) (9663.003). The participants gave written informed consent to participate in the study before taking part.

#### Inclusivity in global research

Additional information regarding the ethical, cultural, and scientific considerations specific to inclusivity in global research is included in the Supporting Information (S1 Text).

#### Study context

This phenomenological study was embedded in a longer-term project, *Siyaphambili Youth (“Youth Moving Forward”)*, which aims to co-create an intervention to address the social contextual factors that create syndemics of health risks for young people living in informal settlements and rural communities in KwaZulu-Natal (KZN) province in South Africa. The project is a partnership between youth peer research assistants (YPRAs), the South African Medical Research Council, a South Africa NGO called Project Empower (www.projectempower.org.za), and University College London.

YPRAs were young people residing in selected communities and were hired to participate as community researchers. Project Empower recruited YPRAs through an iterative hiring process designed to maximise possibilities for vulnerable youth to participate. Preliminary selection criteria included youth who were not currently in school or formal work, and were between 18 and 29 years old. An informal introduction to the project was held in both rural and urban settings. The project responsibilities, ethical considerations and payment terms were discussed in detail over the course of several days before YPRAs were asked to give their informed written consent and were officially hired by Project Empower. YPRAs were hired through South Africa labour law, and thus earned minimum wage and accrued leave and sick leave as per basic conditions of employment. Following this process, 17 YPRAs (9 women and 8 men) joined the project: five urban women, four urban men, four rural women and four rural men. The recruitment period ran from 17 November 2020 to 22 January 2021.

Since the project began in November 2020, the YPRAs have participated in four phases of intervention co-development [further information about the recruitment process and the YPRAs is detailed elsewhere, see 29]. In this paper we use data from the first phase of the study which focussed on understanding the syndemics of HIV, intimate partner violence and poor mental health risks (i.e., how social contexts create overlapping risks characterised by poverty, violence, mental health vulnerabilities, and gender inequalities), and young people’s agency in the face of such risks.

### Data collection

The YPRAs were living in two communities in KZN in South Africa at the time of their recruitment. One group was living in an urban informal settlement in the main metropolitan area, eThekwini, the other group was living in a remote rural community (about 7 hours’ drive from eThekwini), to the north of the province. Both communities had challenges for young people in terms of access to work, precarious livelihoods, and high rates of HIV [30]. Despite being from different parts of KwaZulu-Natal, the two groups of young people were similar in that they were all currently unemployed, out of school and above the age of 18.

Data in this study comes from a data collection process termed ‘artefact creation’, a variation on photo-elicitation, which was done in conjunction with the 17 YPRAs. Between December 2020 and January 2021, we asked the YPRAs to take daily photos/videos (using their phones) to represent their lives and share them with Project Empower facilitators. This is a PhotoVoice project, and as such the photographers / participants gave permission to use their photos for research and related publishing activities as part of the original consent process. There was no particular request to focus on food in this activity. Prior to beginning the artefact creation process we discussed with the YPRAs the ethics of taking photographs and the need to seek verbal permission from participants prior to taking photographs. At the end of this period each YPRA individually identified their ‘top 10’ photos/videos and discussed why they were important to them and the meaning they held, with trained Project Empower facilitators (who form part of the authorship team). The YPRAs led those conversations with only minimal prompting to encourage further reflection and discussion about why they had chosen the photographs to discuss. The facilitators also identified one or two additional photos/videos that they felt were important and asked further questions about these in addition to asking the YPRAs to reflect on important aspects of young people’s lives potentially not captured in their artefacts. These interviews followed an unstructured format and were guided by what the participants wanted to talk about, and the facilitators used a relaxed conversational style and prompts to encourage further reflection on the meaning behind the selected photos/videos. All interviews were recorded using digital devices.

Questionnaires or focus groups can often limit research insights into how people engage with food crises [9], and food more generally. By using photos and videos as an entry point for discussion, we were able to hear stories around food and gender identity that may not have been captured in less artistic means of self-expression; such visual methodologies encourage a richer analysis of meaning making [9].

### Data analysis

Recorded interviews were transcribed verbatim and then translated into English and checked for quality by Project Empower. The transcripts were then prepared and imported into QSR NVivo software to facilitate data coding and analysis. The transcripts were labelled with gender (male/female) and location (urban/rural) to help compare views between different social positions. Guided by the research question, Braun and Clarke’s six steps to conduct thematic analysis were followed [31]. Specifically, an inductive coding process was undertaken, whereby each transcript was critically reviewed (along with the corresponding photos and videos), and any mention of food, drinking water and alcohol were coded into open (descriptive) codes. These were then organised into groupings of similar codes (i.e., axial codes). The underlying quotes for these axial codes and open codes were then reviewed to create a coding matrix in Microsoft Excel with corresponding themes and subthemes [32]. The first author coded and identified quotes to go with each subtheme and all the data were analysed and integrated together in a presentation of initial themes for a group meeting between the first author, second author and last author, which helped to finalise themes and interpretation. While the sample size was small, there was substantial repetition in themes identified in the transcripts and saturation was achieved. The key themes and subthemes are presented narratively and supported by relevant quotes.

## Results

YPRAs collected 710 artefacts (with 61% (n = 435) from the urban YPRAs and 39% (n = 275) from the rural YRPAs and 49% (n = 347) from female YRPAs and 51% (n = 363) from male YPRAs), comprising 58% (n = 413) photos or screen shots and 13% (n = 95) videos, with the remainder made up of voice notes (5%, n = 33) and written text messages (24%, n = 169). Seventeen artefact interviews were conducted, one with each YPRA. Artefact interviews lasted up to 60 minutes each.

Thematic analysis identified precariousness and food insecurity as an important back drop to the social significance of food. To understand the meaning of food and identify spaces for agency in this context, we begin with a description of the constraints young people faced in accessing food. We then focus on the social significance of food. In the presentation of the main themes and subthemes below, participant characteristics are denoted by R/U (to denote rural/urban) and M/F (to denote male/female). Interviewers are denoted by I.

### Precariousness and food insecurity

Food and drink featured in ten of the seventeen YPRAs’ chosen artefacts and all the YPRAs discussed food in one way or another during their interviews, suggesting that food was a prominent feature for all (R/U, M/F) young people’s daily lives, particularly because food was not explicitly asked about when the team described the task to the YPRAs. While food insecurity was certainly common in these communities, no one talked explicitly about being hungry or not having enough food. Instead, the issue of food insecurity or precariousness emerged in more subtle ways as they discussed their artefacts and the meanings attached to the photographs and videos they shared. A central way in which food insecurity was discussed was how young people spoke about the different ways they sourced food, and their problems in securing these food sources. The sub-themes discussed below go into greater detail about how food was a challenge for them and some of the issues they faced.

#### Challenges finding money for food

**Fig 1:**
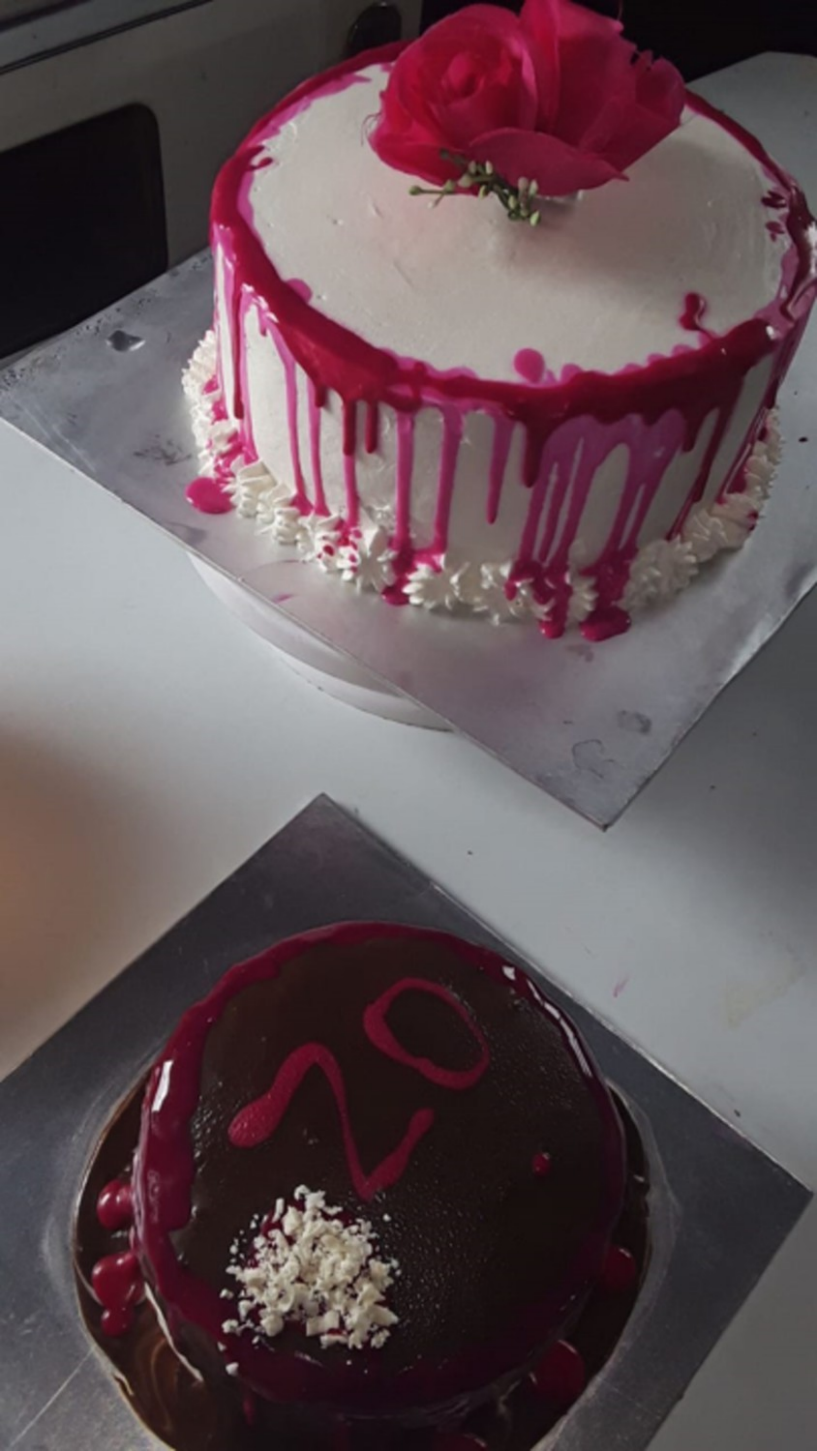
Cakes baked and ready to sell [UF3]. Published under a CC BY license, with permission from the YPRA Photographer, original copyright 2024.

Throughout the YPRAs’ artefacts and interviews they deployed a diverse range of strategies to try and finance access to food. Food was a good motivator to engage in work. Young people from the urban area described how they financed food through odd jobs and diverse income streams, such as sewing [UM2] or baking cakes to sell [UM4, UF3] (Fig 1). However, such work was often poorly paid and insecure, and often they could not earn enough to eat adequately.

Food was also an important incentive or motivator for YPRAs to engage in a variety of activities. A few young people [UM2, RM2, UM4] emphasised how training and employment opportunities could provide food as an incentive to encourage participation in programmes:

“*[We live in] a place of struggles*, *so when you get out there you want to make sure it’s worth your while*. *It can’t be information based*, *it has to include some monetary benefit*, *or vouchers*, *food*, *etc*.*” [UM2]*

Given the lack of resources in these communities, young people described how crime was also driven by the need to obtain money, as described by a young man from the rural area:

*I: What do you think are the reasons for the crime here in the community since it’s not a big place? You know each other…*

*RM3: The reason for the crime in this community is that it’s not easy getting money*.

*I: So, it’s young people who do crime?*

*RM3*: *Yes*, *when a person thinks about money they think about crime*, *they don’t want to work*, *the work they do is crime*, *they want things that will come easy to them*.

Crime may have provided money for food, but some young people also reported stealing food directly as another food access strategy:

*I: Is it a norm that people steal fruit?*

*RM2*: *I also did that when I was still in high school doing grade 9*, *someone had planted grapes and had no seeds and so we were naughty and stole them […]*

Women in the rural area also spoke about how other women relied on transactional relationships with men to secure access to food and money for food [RF3, RF4]. For example, one woman reported that *“to be able to drink or get money they [other women] have to date [employed men]”* [RF4]. While only mentioned in the rural community, the exchange of sex for food and money in transactional relationships likely also occurred in urban areas.

#### Producing own food

**Fig 2:**
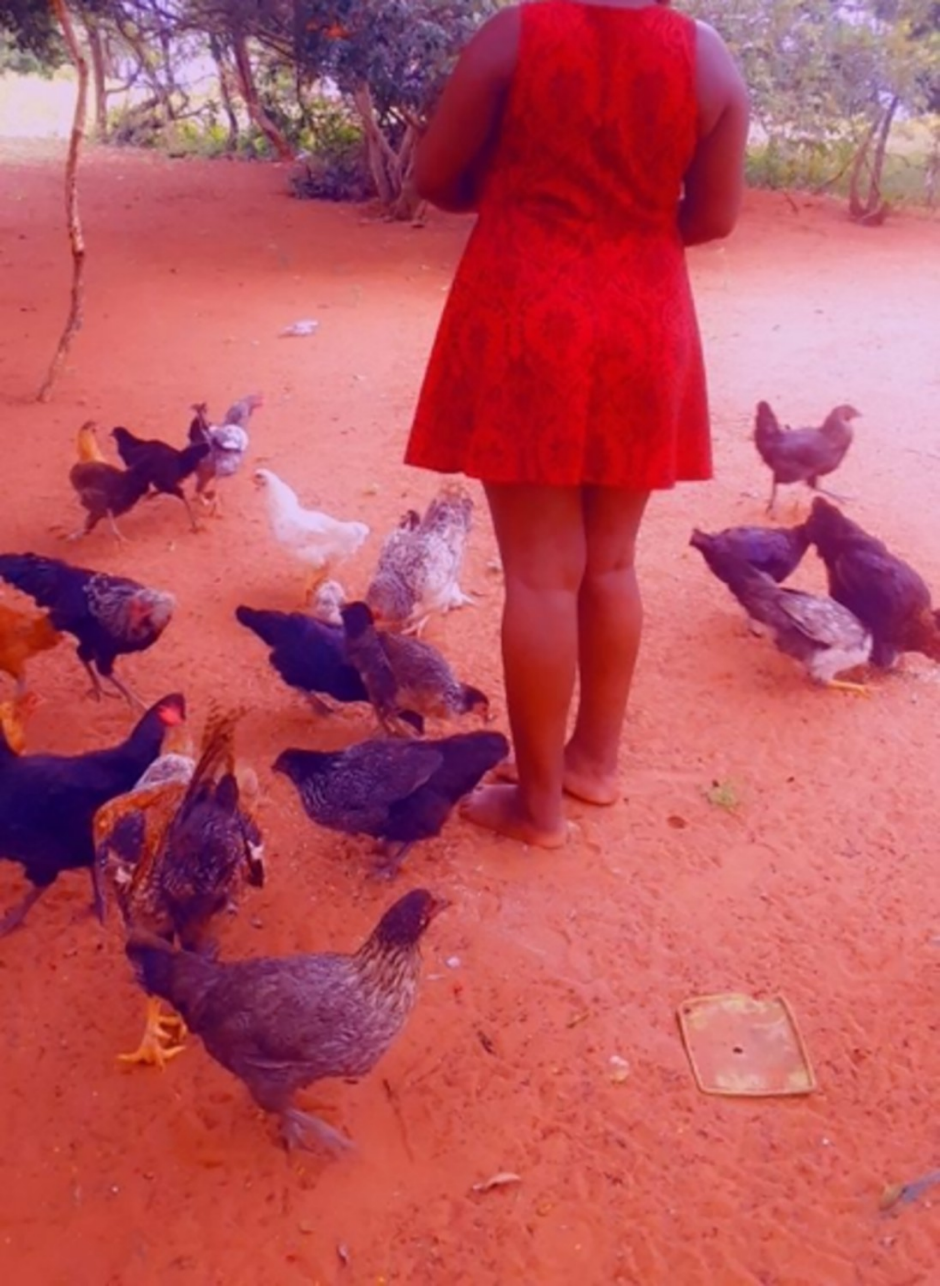
Feeding the family chickens [RF3]. Published under a CC BY license, with permission from the YPRA Photographer, original copyright 2024.

A few of the YPRAs from the rural community described how they produced their own food or were able to source food from their environment. For example, one young man [RM2] described how he and his male friends would sometimes forage for wild fruit in the forest and how young people in the area sometimes planted fruit trees which they took care of with their families. A young rural woman also described helping her family look after chickens at home [RF3] (Fig 2).

As well as these regular food production activities, young people also took advantage of unforeseen opportunities to source additional food, such as fishing after local flooding:

“*The time there were heavy rains*, *rivers were filled […] the fish they got here was able to last them for days because they got a lot of fish*.*” [RF1]*

Young men from rural areas described cattle as a valuable food source [RM2, RM3] with important monetary and social value:

“*Mmmm… as a young person I take livestock as something very important because I can be able to make my family live off it*. *There is a bull and you are able to take it and plant with it*, *you can milk the cows and sell*. *You can even slaughter and sell*, *something like that which will bring in money*. *You are able to pay damages and use the cow if you have made a girl pregnant*. *I take it as something important*.*” [RM3]*

#### Loss of food sources

Young people worked hard to secure access to the food that they wanted, but several challenges impacted on their ability to achieve this. Challenges were linked to both nature and other people, as well as the infrastructure that they required to prepare food.

While people in rural communities were able to produce some of their own food, fluctuations in seasonal cycles sometimes jeopardised this production. For example, whilst the heavy rains brought some extra food for some, they also took away food from others when farming plots were destroyed [RF1]. Crops were sometimes also destroyed by wandering cattle which could create tensions between neighbours:

*RM2: [Laughs] Here is my neighbour’s cattle*, *they are very chaotic as we have a garden full of vegetables*, *these cattle tend to eat our crops*, *so I chase them away*.

*I: How do you deal with these cattle because they seem to be a problem?*

*RM2*: *It’s a problem because I tend to shepherd cattle that I don’t own*, *because they destroy everything in the garden*. *I do that to avoid tension between me and my neighbours*.

**Fig 3:**
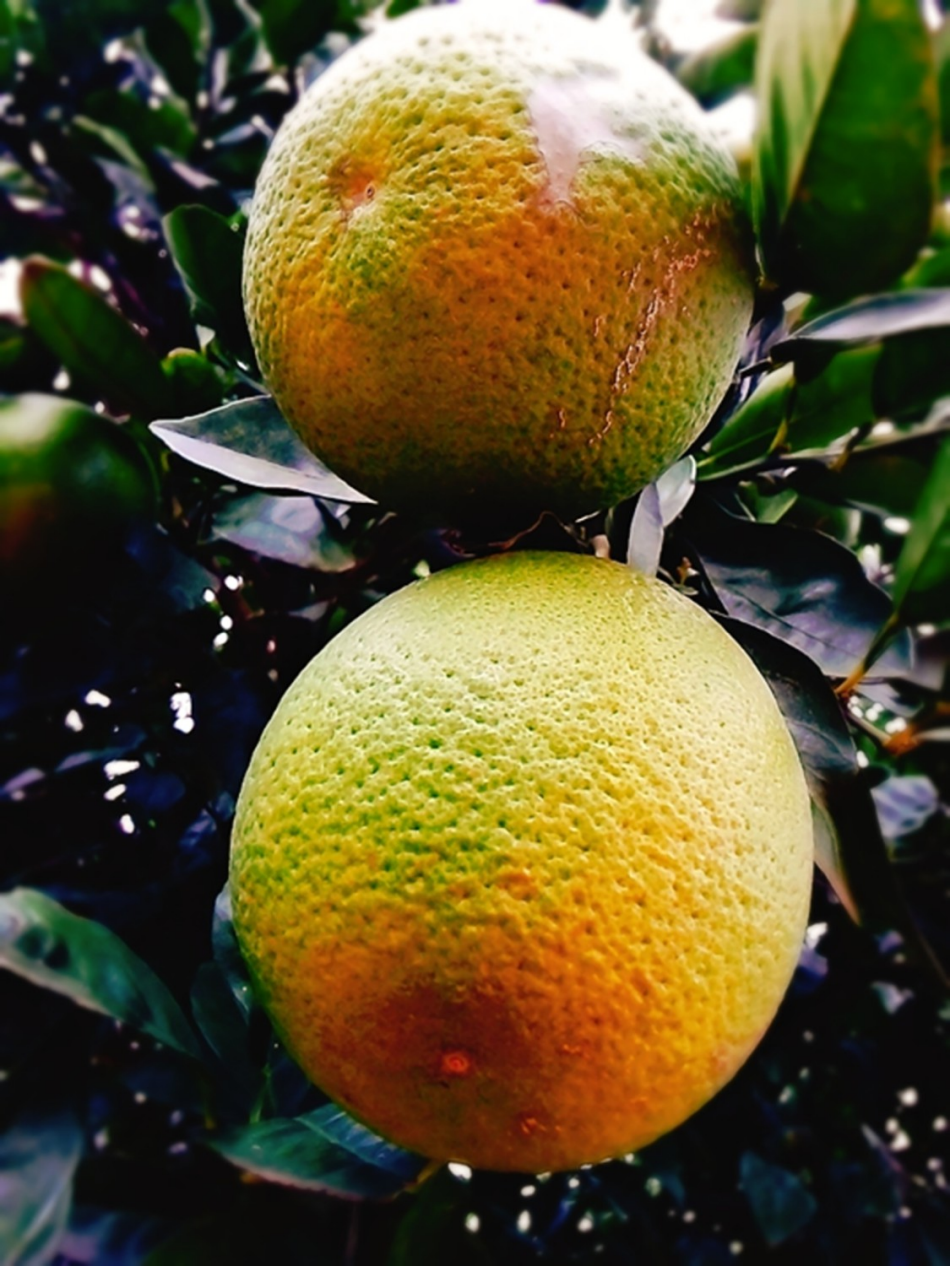
Home grown fruit ripe for the picking [RM2]. Published under a CC BY license, with permission from the YPRA Photographer, original copyright 2024.

While crops were vulnerable to being destroyed by wandering cattle, cattle were also vulnerable to being stolen: *“We used to have cattle when I grew up*, *they got stolen” [RM2]*. Other home-produced food was also at risk of being stolen. For instance, home grown fruit (Fig 3) was sometimes taken by other people:

*RM2: […] we have a fruit garden at home as you can see*, *I took pictures*, *someone came in the night to steal them*.

*I: Ow yah*.

*RM2*: *We only saw footsteps in the morning*, *and you can definitely tell that there was someone else*.

Access to a secure water supply was also a challenge for women in both urban and rural communities (men did not mention this), and this impacted on both their water security and ability to ensure food security (as water was needed for cooking). In rural communities, fetching water was described as a hard chore, often necessitating long walks. In urban settings, the issue was more around water scarcity:

“*Water here gives us problems*. *We wouldn’t have water for the entire day*.*” [UF5]*

*“Hmm we struggle so bad with water this side*, *sometimes we go a whole day without water […] We have got used to the problem of water in our area*.*” [UF3]*

Not only was the water supply itself precarious, but fetching water posed its own risks. As one urban woman described: *“imagine you walk very far to fetch water*, *and someone would bully you and take the water from you when you are close to reaching your home*.*”* [UF5]. And fetching water was especially difficult for urban mothers who were apprehensive about leaving their children with others [UF3, UF5]:

*I: How does it make you feel waking up and there is no water?*

*UF3*: *[It’s] very frustrating*. *Having to take a bucket and going to look for water and that at the same time my son is very young*, *and I have to leave him with someone else*, *yet I don’t like leaving him with anyone*.

Access to electricity was also a significant challenge for YPRAs impacting on their food and water security. During the research period, electricity security was increasingly a problem, with planned and unplanned interruptions to electricity common. The regular electricity outages in the urban areas made it difficult for young people to cook food [UF1], and as one young urban woman described, the lack of power created dangerous situations, including placing households at risk of fire when a candle is used late at night:

“*When there is no electricity and my phone is off*, *it becomes hard*. *I have to wake up and light the candle*. *The time I wake up or when I get out of bed to light the candle*, *the baby would cry in bed*. *I would light the candle*. *I get scared to light that candle because sometimes I would forget about it and fall asleep while I breastfed the baby […] so it’s very dangerous with the candle because it could burn the house and burn us with the baby*.*” [UF5]*

### Social significance of food

Securing food and/or money for food was clearly on young people’s minds, but the meaning and importance of food expanded beyond access to food to include how it was embedded in wider social relationships and the roles food played in these.

#### Bonding through food

**Fig 4:**
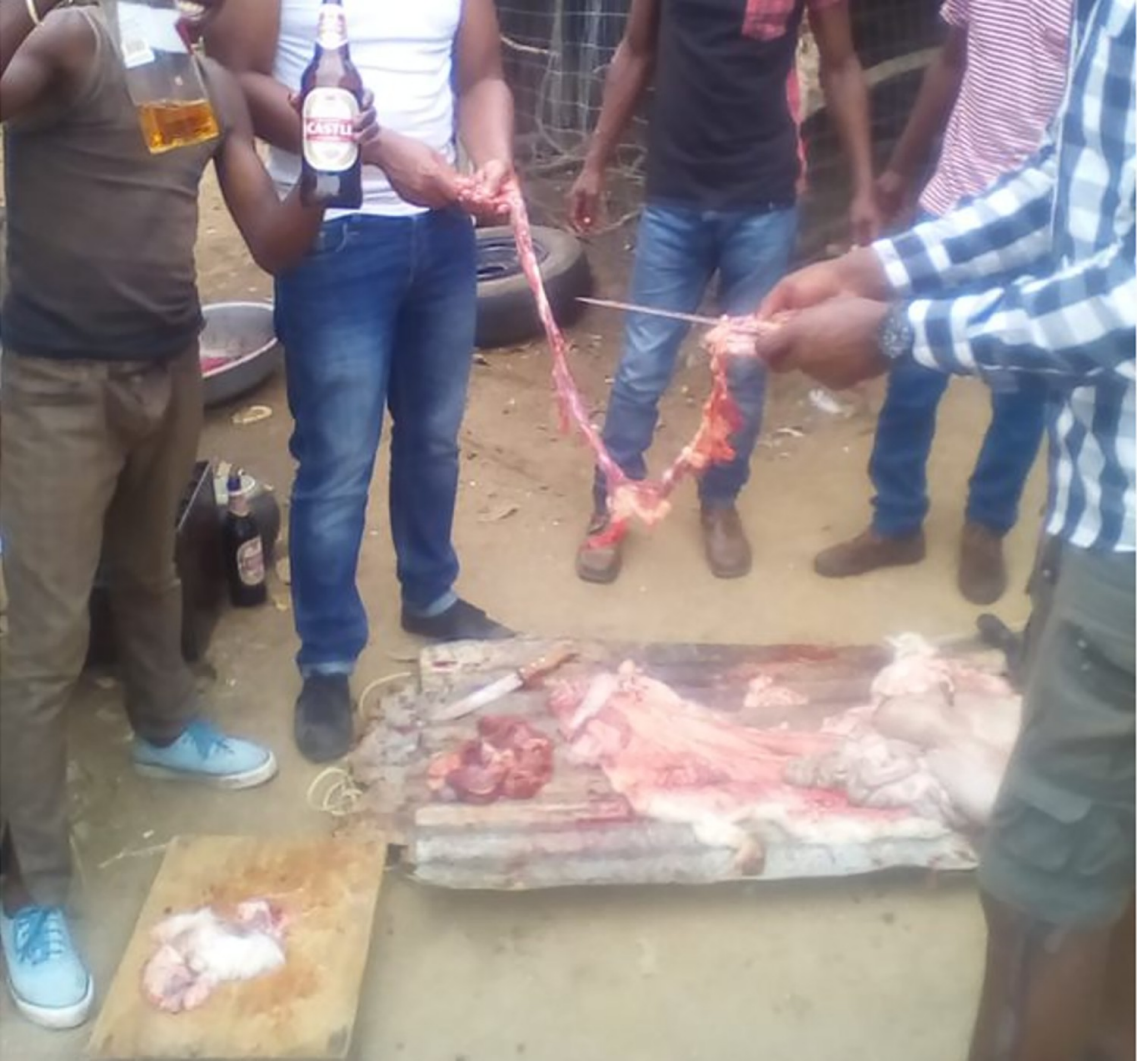
Celebrating the reinstatement of legal alcohol sales with a party [UM2]. Published under a CC BY license, with permission from the YPRA Photographer, original copyright 2024.

Many of the YPRAs talked about the importance of sharing food with each other and how looking to others for food and drink was common. While there were instances when young people ate by themselves, such as when their families were out [RF3] or when they would enjoy special food as a treat alone [UF3], food was often discussed in ways emphasising its role in bonding. Sharing food was a way to celebrate; young people described welcoming back the sale of alcohol (which was banned during COVID-19 restrictions) with a party at a friend’s house with meat and alcohol [UM2, Fig 4], celebrating getting work by eating space muffins [UF3], celebrating passing matric [final year of high school] by going out to eat fast food [RF1], and having a BBQ and wanting to buy a nice cake for a daughter’s birthday [UM2].

Sharing food was also a means to (re-)connect with friends and family. One young woman described sharing recipes in a WhatsApp group with other women [RF3]. Many young people described sitting around eating and drinking with their friends [UM3, UM4] and while eating out with friends was a rare treat for some [RF3], others ate out with their friends often [UM3]. Several YPRAs described the importance of food for connecting with family members. One young urban man described how cooking was a way to facilitate bonding between mother and son and that he would sometimes choose to eat at home to spend more time with his family [UM3]. Women from the rural areas also described how going out to eat with family members also helped to strengthen relationships [RF4, RF1]: *“I was happy that I am going out with my mother and for her to buy us things*, *eat and drink*.*” [RF4]*

Food also played a role in young peoples’ romantic relationships. One urban woman described how she liked cooking with her boyfriend and how she thought this helped to strengthen their relationship, and was potentially signifying a more serious and committed relationship:

*I: How did you feel cooking for your boyfriend and his family?*

*UF5: It was only him and his child. I would go cook for both of them*.

*I: Oh, okay. You didn’t have a problem that you were there to cook?*

*UF5: No, I didn’t have a problem, in fact I was happy*.

*I: What made you happy?*

*UF5*: *[laughs] That I am going there*, *and I will be with him and cooking while he is there with me*, *helping out*.

For other young people, tradition and celebration was embedded in the importance of sharing food. For example, one young man from the rural community described how family members cooked for house guests to show good hospitality [RM1] and a woman from the urban area explained how cooking for her boyfriend’s family and eating together is an important part of the local *lobola* bride wealth payment marriage traditions [UF5].

#### Solidifying friendships through sharing food

Given the challenges related to accessing money and food, young people often shared food and costs, which facilitated further bonding and strengthening of relationships. Young people often clubbed together to buy food and drink and split the costs [RF1, UM4, UM2], but some young people would also willingly pay for everything if their friends did not have any money to contribute on specific occasions [RF4]. Young women described how they spent money on food and drinks for each other [UF2], while young men described how they brought alcohol to each other’s parties [UM2] and bought drinks for others at taverns [RM2]. Sharing alcohol between friends seemed just as important as sharing food, and drinking alcohol was described by young people as something that makes them happy and allows them to relax, and was therefore deemed worth contributing money towards [RF4].

**Fig 5:**
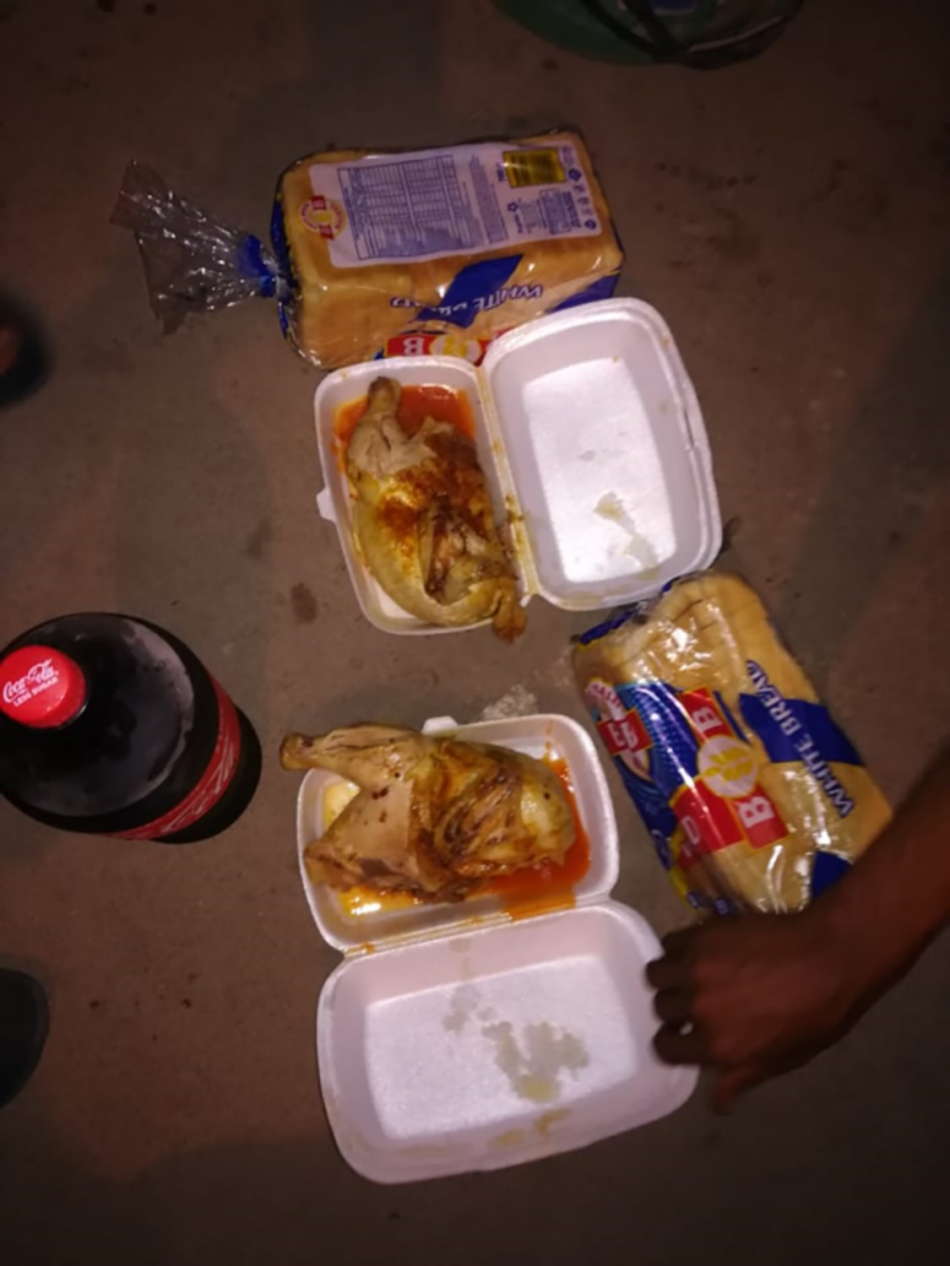
"What we are doing shows working together" [UM3]. Published under a CC BY license, with permission from the YPRA Photographer, original copyright 2024.

The importance of sharing food and drinks to build relationships was clearly described by one urban male YPRA who emphasised how his group of male friends shared food equally, regardless of who paid, treating each other “like brothers” (Fig 5):

“*Let’s say I have a R100 and I’m hungry*, *I need bread and I am with the guys*, *so I need to think they might be hungry too as we have been sitting here*. *So*, *I need to decide to buy something that we can all share*, *if we are having two slices*, *we will all have two slices then we will see what happens […] we treat each other like brothers*.*” [UM3]*

He also explained that eating together “shows working together”:

*UM3: We decided that each person would go home and ask them [family] not to dish for us*, *then come back and eat here*. *We then bought bread and other things as we are eating as friends*. *We bought two breads and full chicken*.

*I: Ok, so eating together as guys…what does this mean to you or to your friends?*

*UM3: I always tell them that what we are doing shows working together…if we keep working together like this until we get old, it would be a good thing as none of us would suffer or face a problem while we are there to help him, because a person is a person because of other people. Because you can’t do things alone and keep saying I have to do this alone*.

*I: So, you all have one goal of working together for it not to end here but to continue even when you have your own families?*

*UM3*: *It should be like that*, *for one to always know that if they need help there is so and so who will definitely help me*.

Sharing of food wasn’t limited to same age and same gender groups. Young people sometimes contributed to buying food for younger people (but avoided drinking together) [UM1], and young men would often share their food with female friends:

“*We actually treat girls in our neighbourhood as our friends…something like that*, *there are plenty of girls that we chill with*. *[…] Maybe we buy something to eat*, *and they would eat with us at that time*.*" [UM3]*

In each of these instances, food and drinks provided a way to build relationships in contexts where there was often a lack of trust and friendship, and where because of hardships, survival relied on support from one another. Relationships were forged as unbreakable bonds that helped to ensure that men were always able to eat, in a context where going hungry is an ever-present threat.

#### Food as signifier of social status and gender roles

Food consumption and provision was also a signifier of social relationships and inequalities, particularly around gender roles.

*Men expected to provide food for families*, *women and children*. A dominant theme throughout the interviews and artefacts was the assumption that men were expected to buy food for households they were part of, and for their girlfriends. In both the rural and urban areas, men were expected to provide for their partners. While none of the men involved in the study were married, they recognised that being in a polygamous marriage would mean having to provide for everyone equally, as one rural male explained:

“*Whatever he does to the other side*, *he should do it on the other side the same way*. *Because if the woman on this side you buy 5kg braai back and on the other side you don’t do the same*. *That will create arguments […]—you have to balance everything you do”*. *[RM3]*

Another young man from the rural areas explained men are often the breadwinners and their death has economic repercussions for families [RM2]. Yet it was the urban male YPRAs who described a much stronger pressure to *“put food on the table”* [UM2] and a sense that cohabitation was a burden [UM2] given the additional pressure it placed on them to always provide for an additional person.

Throughout discussions of the pressure to provide for families or partners, two urban male YPRAs also expressed frustration and disappointment that their partners didn’t understand the pressures these expectations put on them, or that when a man was unemployed the expectations were unrealistic:

“*…I was disappointed in her because she should understand I don’t have anything to put on the table*, *but she just takes whatever I give her […] the pressure was that all the things that I could provide for her*, *I could not anymore because of being unemployed*. *So*, *it was hard for her to adjust*.*” [UM1]*

“*As a guy you also want to impress her cause she’s got friends […] she’s the one who puts pressure*, *but you find that she is not aware*. *[…] I don’t have money so she can’t expect that she will get something […] that’s one thing I don’t like*, *for her to have expectations when coming to me…that if she is with me*, *she will get this*, *this*, *and that*. *[…] The fact that I am weak when it comes to money*, *I would end up under pressure just to be able to make her happy*.*” [UM4]*

Men were also expected to provide food for children, whether or not they were still in relationships with the child(ren)’s mother(s). They lamented that baby formula was expensive [RM3] and described childcare support as another burden they faced [UM2]. Furthermore, the inability to provide such support was described as leading to conflict with the mother of their child:

“*…when you are struggling*, *you get the insults that you are not maintaining your child*, *and [you both] keep insulting each other”*. *[UM4]*

However, men described trying to contribute the little that they had [UM2, RM3], and food was seen to express fatherhood and love, even when they had little money:

“*…you don’t have to be rich to be able to play a role to your child*. *You don’t have to have a lot of money*, *even if you have R20*, *if she [his child] wants R1 chips and you’re able buy them*, *that’s already a role*, *you were able to play a role of being a father” [UM4]*

**Fig 6:**
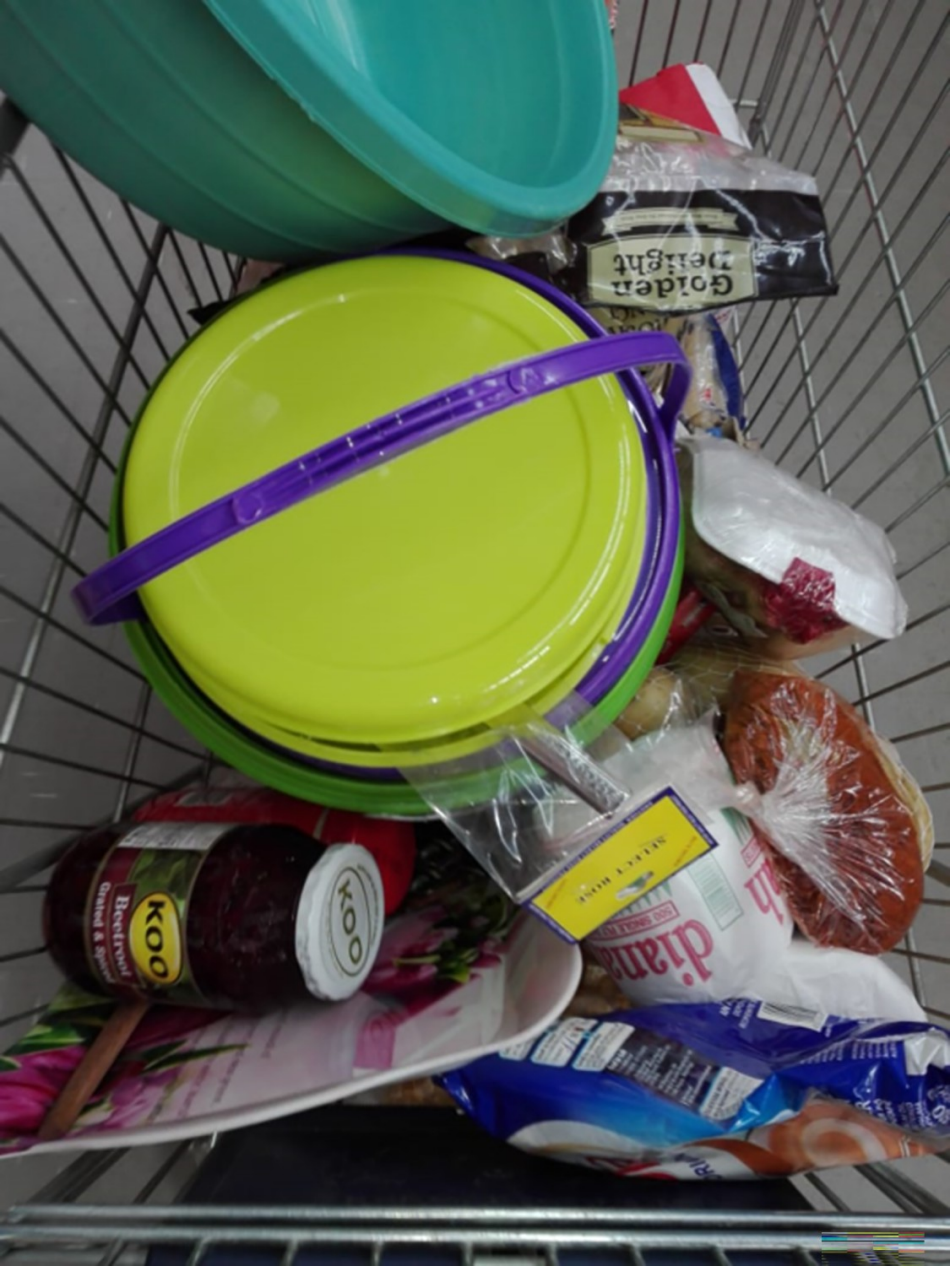
"A big burden has been removed" [UM4]. Published under a CC BY license, with permission from the YPRA Photographer, original copyright 2024.

Given the struggle most men described in trying to provide for their families, partners, or children, when they could provide food, they felt great relief and a sense of achieving what was socially expected (Fig 6):

“*It had been a while since I bought everything that I needed […] So*, *I was feeling alright as if there’s something that was lifted off my shoulders*, *a big burden had been removed”*. *[UM4]*

*Men buy food and drink to demonstrate status to other men and to impress women*. For men, being able to buy food and drink for other men and women, was something that they sought to do to achieve a sense of respectability, which was closely tied to being able to work and the idea of being more successful:

“*In communities we live in*, *if you have something you are listened to*, *but if you have nothing*, *they say what will you tell us while you have nothing*.*” [UM3]*

For some men, they explicitly linked being able to buy food and drink in public settings to a certain sense of masculine satisfaction:

“*I think you see a real man from a distance*, *[you see] that this is a real man*, *this usually happens in taverns*. *You know when you arrive in a tavern with R500 and you find other guys drinking*, *just buy maybe four beers and give it to the guys*. *You find girls eating food*, *just buy food for them*.*” [RM4]*

Men tended to be the ones responsible for supplying alcohol at parties [UM3] and male YPRAs described how men tended to spend more money on alcohol when women were around:

“*… most guys have that tendency that if there are girls we should drink then […]*. *But here you wake up in the morning with a hangover then go to the fridge to get a can of cold beer and drink just like that*, *there’s no stress that there is money that you overused*. *Others went to withdraw money at night when they ran out of alcohol*, *nothing like that happened here*. *But if there were girls involved it was going to happen”*. *[UM4]*

Buying food and drink was also an important way for men to demonstrate love and affection towards their girlfriends. Men described this as spoiling their girlfriends [UM3], and they also sometimes bought drinks for their girlfriend’s friends [RF4], which may have been a way to further impress them, although girlfriends tended to get special treatment compared to other women:

“*If we are drinking Amstel [beer]*, *they drink what we drink*. *Unless one of us brings their girlfriend*, *then that person buys her whatever she likes to drink” [UM2]*

However, these gifts weren’t always cost free. As many young people explained, men sometimes expected sex in return for buying women drinks. Whilst this was sometimes described as a mutual understanding (e.g., *“I guess they had an agreement because we’ve never had a case where they say someone was beaten up because she didn’t want to sleep with him”* [RF3]), it also sometimes led to the fear of, or actual, violence:

“*There was that fear because sometimes he [a friend’s boyfriend] would bring his friends for us whereas we are not part of his plan*. *So that is also a risk because you don’t know when a person is going to show his true colours*. *We are already drinking their money so after drinking their money*, *they obviously expect us to go sleep with them”*. *[RF4]*

“*But of course*, *there are those that when they buy a girl a drink they do so with ulterior motives*. *They start forcing the girl to go home with them*. *But that happens everywhere […] you don’t drag her by force but the thought to make it clear that she must give something in return does come to mind”*. *[UM2]*

*Women are expected to cook*. YPRAs highlighted the expectation that women were meant to cook, and this social norm was particularly common in women’s discussions. Their interviews and artefacts made it clear that women played a central and socially significant role in food preparation. For example, men asked girlfriends to cook for them:

“*I would go to him while he was my boyfriend while his mother was not home*, *he would ask me to cook for them food that could last for the entire weekend*. *I would go and cook for them*.*” [UF5]*

**Fig 7:**
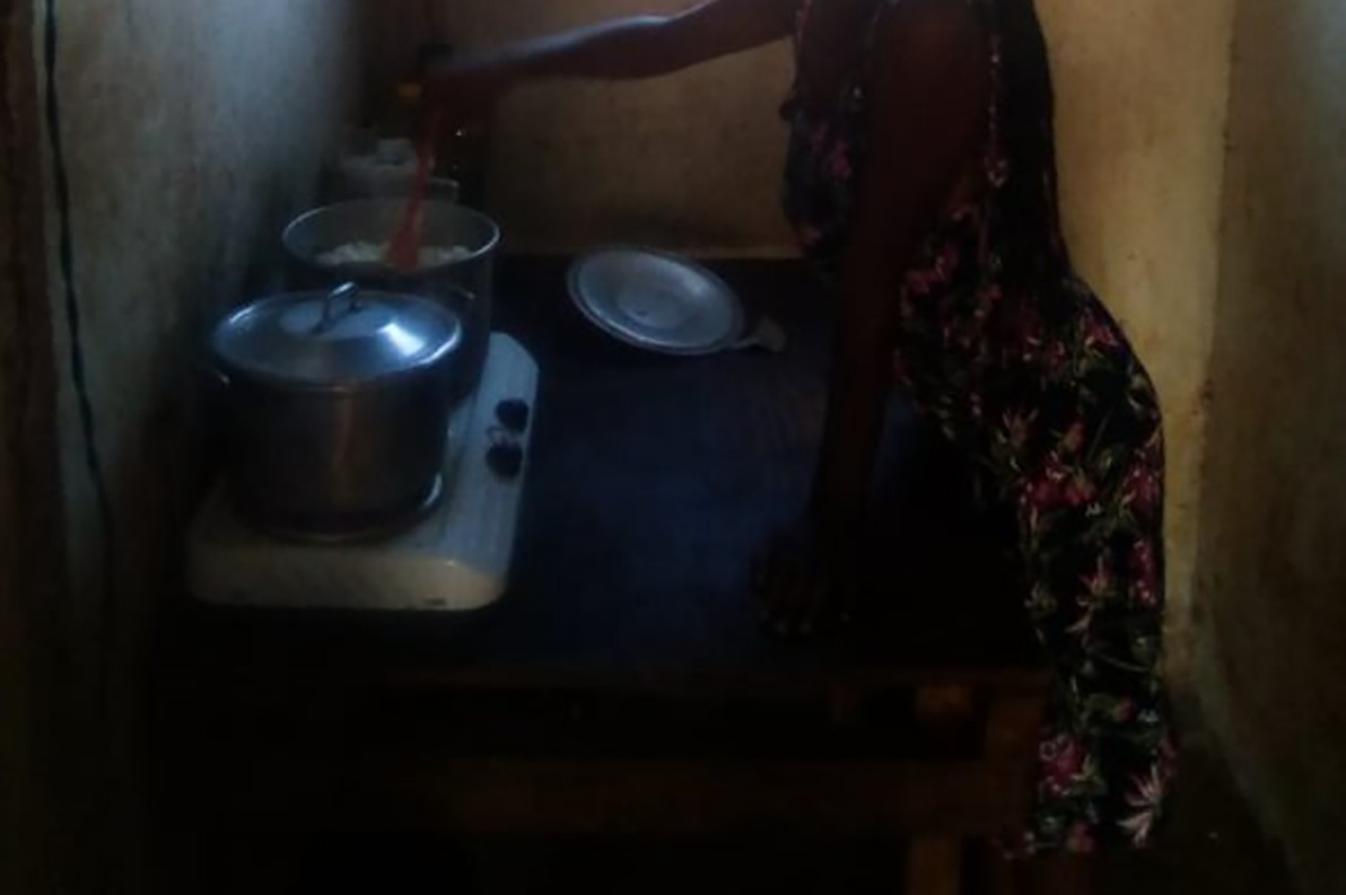
"I enjoy cooking" [RF1]. Published under a CC BY license, with permission from the YPRA Photographer, original copyright 2024.

And once married, women were expected, rather than asked, to cook for their husbands and in-laws [UF5]. Opinions on women’s obligation to do the cooking were varied. Some girls enjoyed cooking at home (Fig 7):

“*I enjoy cooking*. *[…] I have no problem with cooking*, *I can even cook for the whole year*. *I really don’t have a problem with cooking*.*” [RF1]*

While others resented that they were expected to cook all the time:

“*Boys cook when they feel like it […] But a young girl doesn’t have a choice*. *[*..*] It doesn’t sit well with me*. *[…] I do that when I am forced to*. *[…] Others complain*.*” [RF2]*

Whilst girls were expected to cook and clean, the load was often shared between sisters who took it in turns to do these chores [RF2, RF4]. Some girls refused to cook, but still helped out with other chores [RF4]. One woman described how girls taught younger family members how to cook:

*“I [usually cook at home] and my sister’s child sometimes [helps]*, *but it’s the same as I am the one cooking because she is still learning*, *so when she is cooking*, *I need to be there*. *Her too*, *but I am the one who cooks the most*.*” [RF3]*

Although norms around women’s responsibility for food preparation were evident, both male and female YPRAs did mention that some men did cook [RF1], although this was described as usually only out of necessity [UM3]. Men also explained how they would only cook simple meals as they were too busy, as described by one urban man below:

“*As a guy…I don’t know what the reason is that even when you have everything*, *everything that you wish to do but you are always busy*. *[…] when you are about to start putting rice on the stove then someone comes and you have to leave*, *he would be like “please help me with this”*. *[…] and you are hungry at the same time you want to eat now*. *[…] I was doing something fast […] I was busy and didn’t have time to spend being busy cooking”*. *[UM4]*

The same man suggested that men would excuse themselves from cooking for girlfriends, explaining that *“She can’t expect me to cook […] I can’t cook […] I sometimes cook food that I can’t eat*!*”* [UM4].

But not all men couldn’t/wouldn’t cook and young people from the rural areas commented on how upbringing likely affected gendered attitudes towards cooking:

“*They [boys] do cook sometimes even if the girl is good to cook*, *they cook and sometimes wash the pots and go cook*. *I think it’s how they were brought up*.*” [RF3]*

“*Sometimes it’s how you are brought up by your family*. *As I grew up with females*, *they will sometimes tell you that they will not cook” [RM2]*

## Discussion

Among young women and men in two communities in quite different settings of KwaZulu-Natal, South Africa, but facing similar overlapping challenges related to high rates of poverty and unemployment, as well as social marginalisation, the issue of food emerged in data collection in quite similar ways. Accessing and securing food was challenging and shaped by contexts often outside young people’s control. The YPRAs described a range of strategies to try and secure food. Only in rural communities was direct production (e.g., foraging for food and farming animals) a practical option (although constrained by external factors including crime and climate change). For most YPRAs, in both the rural and urban areas, earning money through work was the only meaningful strategy. However, the macro contexts that young people lived in constrained these food procurement strategies. Furthermore, discussions of water and power supply issues highlighted how inadequate infrastructure posed an additional structural threat to food security [11].

Similarities in findings from the urban and rural settings may be explained by similarities in the broader social structures that limit food access for young people in both contexts. Food insecurity in rural areas has been identified as a significant global challenge, particularly in areas where many people rely on small-scale farming for their livelihoods [3,33]. Meanwhile, rapid urbanisation, the loss of agricultural land, and reliance on supermarkets, smaller traders and corner shops for food contributes to food insecurity in South Africa’s urban informal settlements [9]. Although such differences contribute to unique factors that in turn contribute to food insecurity in urban versus rural settings, the interconnectedness of macro, meso, and micro factors [11] are relevant in both settings. Food, water, and energy security are intrinsically linked and secure access to both water and energy are needed to ensure that people have access to sufficient, safe and nutritious food [34].

Food sharing among friends was a common experience described by YPRAs, providing a way to build relationships with one another. Such cooperative behaviour may be an important way of expressing agency in these food insecure contexts. Previous research by anthropologists has highlighted how sharing a meal is about the creation of kinship [35], but can also signify division [12], as food sharing is not indiscriminate, but rather is done selectively to build and maintain different types of relationships. Sharing food with friends was described by young urban and rural men and women alike. The social significance of food was most pronounced in the accounts of young urban men in our study, in contrast to other South African research which suggests that changing values of food may lead to less sharing in urban areas compared to rural areas due to increased material needs [16]. The urban male YPRAs also talked about sharing food equally and treating each other “like brothers” and said that buying and sharing food is seen as a symbol of men working together. The reality is that for these young men living in precarity, emotional bonds and the provision of food are indivisible. Given the social disintegration in both the urban and rural communities [36] and the relative lack of social capital in these settings [37], YPRAs actively seeking to establish alternative relationships outside of the households and build peer communities through the use of food. This demonstrates important agency and an effective way to buffer against food scarcity in these contexts. Food also had other meanings as well, linked to establishing power and identity in a group. As such, the act of sharing food, while intensely important in building and sustaining relationships in contexts of social disintegration, was also important to establish relationships of hierarchy in groups.

Food and provision of food produced and reproduced power inequalities [10], social status, competition, and agency. There was a strong normative assumption about men’s need to provide food in general, and specifically to their male friends and their girlfriends, which established identity and power hierarchies in these contexts [9,10,38–41]. Food sharing wasn’t simply a prosocial act, as sharing food and drink seemed to garner status amongst men, and YPRA accounts revealed that who was witnessing this sharing was also important in shaping social status [38,42]. Consumption of any food and drink in social spaces may indicate improved socioeconomic standing, but the consumption–and especially the sharing–of high value items like meat may be a particularly vivid symbol of relative wealth and generosity [43]. Similarly, women’s provision of food through cooking for boyfriends/family was also embedded in a similar but different way in the establishment of social identity.

Therefore, food can serve as a means of cooperation and social support, while also constructing and reinforcing social status competition and individual agency. Gender is key to power relations and the intersection of food and gender highlights the ability of food to construct the self in relation to others, materially and symbolically [10]. Men striving to improve their hierarchical position through food is implied in the gender role beliefs evident in our data, with men’s ability to provide food to others contingent with gendered expectations of them as breadwinners. While the masculine breadwinner ideal positions a man as a provider not only of food, but of shelter, safety and security as well, the young men in our study, had to rely on the provision of snacks and treats for a child or an intimate partner as a symbol of their masculinity, rather than being able to fulfil wider expectations.

Gendered expectations around food also applied to women. Across this study, young people talked a lot about how women were expected to prepare food for partners, children, and family members. Generally, across cultures, women and girls play a crucial role in food provisioning and preparation and the “kitchen-space” provides a location in which to perform female gender [10,35]. Several of the YPRAs described how women and girls were expected to cook, with the teaching of younger family members demonstrating an element of cooking creating women, resonating with anthropological studies from other African contexts [35]. Whilst young women were living in the same precarious conditions as their male peers, and often struggled to access sufficient water and electricity to prepare meals, they were still able to perform the food preparation roles that affirmed and reinforced their femininity.

Our findings point to the specific ways in which food can construct both masculinity and femininity [12], and how gender is performed through food production, with men expected to provide, and women expected to prepare meals [10]. As the discussions with YPRAs highlighted, these expectations can on the one hand create pressure in already stressful environments [44], but they also allow innovative expressions of agency to emerge. For example, food can be used as a form of self-representation and ’feminine talking’ through the connection of food preparation with feminine identity [12]. In our data, young South African women described the preparation of food for those they cared for in ways evoking the love language of acts of service, and inferred positive ramifications for their relationships [45]. Rather than feeling oppressed by these gendered expectations (in general), women were able to express agency through cooking for their loved ones [10,35]. Similarly, food also provided a means of ‘masculine talking’, where masculinity was constructed through the representation of sharing food to foster status in YPRA’s descriptions.

Logically with such interconnections between food and gender identity, we might expect there to be ramifications of food insecurity for gender-based violence. Whilst correlations between masculinities, food insecurity, and intimate partner violence are well established [46–48], our study did not find any explicit connections made between lack of food and violence. We did however witness implied and sometimes overt discussions about the interconnections between the provision of alcohol by men to women, and the expectations around sex this fostered, as well as the sometimes violent consequences if these expectations were not met. This reinforces a large body of research which has similarly described how men’s provision of alcohol is implicitly linked to expectations of sex [49,50].

Central to feminist intellectual and political work is the idea that gender is always related to other social identities [9] and an intersectional lens highlights that even within our two study sites, the varieties of experiences with–and relationships to–food expressed by the YPRAs is testament to food expressing social hierarchies. Intersectionality recognises food as a site of struggle and social, individual, and creative struggles are crucial in connecting struggles over hunger to broader struggles and systems of meaning making [9]. Our data showed some urban versus rural differences in relation to food security, highlighting how gender intersects with other social identities such as those in relation to geographic location. For example, urban men talked about food the most, perhaps suggesting food insecurity was a more pressing issue for this group who tended to have less support from family, and for whom status displays may be particularly important [41].

### Limitations

The recruitment process for the YPRAs while trying to focus on vulnerable youth may have led to bias through exclusion of some youth, such as those with disabilities, or those with caring responsibilities. As such, this was a partial set of insights into young people’s lives. During analysis, the potential limitations of the lead author’s positionality and relative privilege as a white UK-based academic woman from a completely different context may have influenced the interpretation of the data. To mitigate against this, the lead author regularly reflecting on personal biases and assumptions during data analysis, seeking input and feedback from colleagues with diverse backgrounds and perspectives, as well as sense-checking themes with the broader literature on the meaning of food. This helped to ensure that the research was conducted and reported in a way that was ethical, transparent, and respectful of the participants’ experiences and perspectives. Moreover, while the aim of qualitative research is to understand meaning and/or experiences of social phenomena, we also recognise that the findings may not be generalisable to other regions of South Africa or to other countries where understandings of food and dynamics of food insecurity may be considerably different.

## Implications and conclusions

Our study has shown the crucial role that food plays in shaping young people’s identities and social networks in urban informal settlements and rural villages in KwaZulu-Natal. Beyond nutrition and sustenance, food also carries significant cultural and social meanings that influence young people’s perceptions and experiences of food insecurity, and our findings describe the social meanings associated with food insecurity for young people living in KwaZulu-Natal, South Africa.

Understanding how young people perceive food and experience food insecurity can inform the design of culturally appropriate and community-led food security interventions. Our findings have important implications for future interventions to address food insecurity in demonstrating the diverse and complex ways in which young people’s identities and social networks are linked to the social meanings of food. Interventions are needed that address these complexities by nurturing young people’s efforts to share food and ensuring that the food they share is nutritionally beneficial. The quality of the food that we consume can be emphasised as part of behaviour change programming.

The findings of this study also have important implications for food security measurement and policy development. Firstly, incorporating sociocultural factors such as gender and other power dynamics in the measurement of food insecurity can provide a more accurate and nuanced understanding of food security issues, and help to operationalise food as a social, and not just economic, commodity. Secondly, acknowledging the social significance of food in policy development can lead to more comprehensive and effective interventions that prioritise the empowerment of local communities.

## Supporting information

S1 Text PLOS’ questionnaire on inclusivity in global research. (DOCX)
